# Eight weeks of core stability training improves landing kinetics for freestyle skiing aerials athletes

**DOI:** 10.3389/fphys.2022.994818

**Published:** 2022-11-03

**Authors:** Ming Wei, Yongzhao Fan, Zulei Lu, Xuesong Niu, Hao Wu

**Affiliations:** ^1^ Capital University of Physical Education and Sports, Beijing, China; ^2^ School of Sports Training, Shenyang Sport University, Shenyang, China; ^3^ School of Kinesiology and Health, Capital University of Physical Education and Sports, Comprehensive Key Laboratory of Sports Ability Evaluation and Research of the General Administration of Sport of China, Beijing Key Laboratory of Sports Function Assessment and Technical Analysis, Beijing, China

**Keywords:** freestyle skiing aerials, core stability, landing, performance, body shape

## Abstract

Freestyle skiing aerials are characterized by technical elements including strength, flexibility and balance. Core stability in aerials can improve sporting performance. Objective: This study aimed to analyze the effect of 8 weeks of core stability training on core stability performance in aerials. Methods: Participants were randomly assigned to a control group (CG; *n* = 4male + 5female; age 15.89 ± 1.54 years; height 163.11 ± 6.19 cm; weight 55.33 ± 5.07 Kg) and a training group (TG; *n* = 4male+5female; age 16.11 ± 2.47 years; height 161.56 ± 5.25 cm; weight 57.56 ± 8.11 Kg). Body shape, the performance of core stability, and landing kinetics were measured after 8 weeks of core stability training. Independent sample t-tests were used to compare baseline values between groups. A two-way repeated-measures analysis of variance (ANOVA) (time × group) was used. Results: The TG improved body shape, and waist circumference (t = −2.333, *p* = 0.020). Performance of core stability, squat (t = −4.082, *p* = 0.004), trunk flexion isometric test (t = −4.150, *p* = 0.003), trunk lateral bending isometric test (t = −2.668, *p* = 0.008), trunk rotation isometric test (t = −2.666, *p* = 0.008), side bridge (t = −2.666, *p* = 0.008), back hyperextension (t = −4.116, *p* = 0.003), single foot triple jump (t = −4.184, *p* = 0.003), and single-leg balance with eyes closed (t = 4.167, *p* = 0.003). Performance in landing kinetics, End/Phase (t = −4.015, *p* = 0.004), sagittal axes (t = −4.598, *p* = 0.002), frontal axis (t = 3.116, *p* = 0.014), peak power hip changing range (t = 2.666, *p* = 0.017), peak power knee changing range (t = 2.256, *p* = 0.049). Conclusion: Core stability training leads to improvements in body shape, the performance of core stability, and landing kinetics. Therefore, these improvements can improve the sporting performance in aerials competitions.

## Introduction

Freestyle skiing aerials (aerials) began in the 1960s and became an Olympic sport at the 1944 winter Olympic Games in Lillehammer. Aerials are like gymnastics on snow, stability, difficulty, accuracy, and grace are distinct characteristics of aerials. Elements of physical fitness such as spatio-temporal perceptual ability, nervous system control capability, core stability (CS), centrifugal contraction ability of lower limbs, balance, and coordination are determinants of performance in aerials (Niu, 2010). Therefore, physical, technical, and mental skills, motor control, and harmony of movement are key elements in the performance of aerials.

Since the 24th Beijing winter Olympic Games, the technical difficulty of the aerials has increased significantly. Male athletes mainly focus on triple movements, and the commonly used movements include bFFF (4.050), bFdFF (4.425), bdFFF (4.525), bFFdF (4.525), and bdFFdF (5.000), especially the ultra-high difficulty movement bdFFdF (5.000) has become a necessary movement for the championship. Female athletes showed a tendency to develop from double movements to triple movements, with the commonly competed movements changing from bFF (3.150), bdFF (3.525), bFdF (3.525), bdFdF (3.900) to bLTF (3.500), bLFF (3.800), bFFF (4.050), bLdFF (4.175). Ge (Bingzhu and Ming, 2001) statistically analyzed the World Cup competitions routines of women’s freestyle aerials from 11 countries and found that the success rate decreased as the technical difficulty increased. According to the FIS aerials judge’s manual scoring standards, the air score accounts for 70% of the total score of the competitive competition, the landing score accounts for 30% of the total score given by the judges, but the success of the landing action is more intuitive than the air action. Thus landing stability is also an important part of the quality of the action, which is the final score of the deciding factor.

Landing failure will not only affect the total score but also cause sports injuries. For example, the rate of knee joint injury in aerials athletes on the Chinese national team is close to 85% and higher in retired athletes (Fu et al., 2019). Studies have shown that instantaneous impact in the vertical direction will damage the cartilage of the knee joint, while the long-term repeated impact will cause strain damage to the stress concentration region of the cartilage (Meng et al., 2017). Other studies have shown that when the knee joint flexes at a certain angle, shear or torsion stress caused by instantaneous movement of the tibia may damage the cruciate ligaments (Xie et al., 2009; Atarod et al., 2013), while instantaneous inversion or eversion of the knee may cause the medial or lateral collateral ligaments to be damaged (Gardiner and Weiss, 2003; Luetkemeyer et al., 2018). In addition, the athletes’ ankle joints are essentially locked in snowshoes, which do not provide sufficient cushioning at the moment of landing, resulting in greater impact force to be absorbed by the knee joints.

There are some evidences that core stability training (CST) is effective in improving landing performance (Ford et al., 2003). Dynamic stability of the trunk and lower limbs are based on the neuromuscular control of the lumbo-pelvic-hip complex. This complex consists of the hip, pelvis, and trunk segment, as well as the muscles that cross these joints (Hibbs et al., 2008; Okada et al., 2011; Oliver et al., 2012). Associations between poor core stability of the trunk and non-contact anterior cruciate ligament (ACL) injuries in female athletes (Leetun et al., 2004; Hewett et al., 2006; Zazulak et al., 2007). Specifically, poor core neuromuscular control may increase external hip adduction and knee valgus moments during landing (Leetun et al., 2004) which increases ACL loading (Shin, Chaudhari, & Andriacchi, 2011). CS is important to athletes and recreationally active individuals alike as it provides proximal stability for distal mobility, especially in cases involving spinal stability (Panjabi, 1992; Akuthota et al., 2008). Furthermore, Myer (Myer et al., 2006) reported that a neuromuscular training program that included both balance and mainly dynamic core stability exercises for the trunk and pelvis significantly reduced impact landing forces, whilst plyometric training did not. While this evidence suggests incorporation of CS exercises into a training routine can reduce peak landing forces and may also lead to a decrease in injury risk, it is unclear what the specific impact of a trunk dominant CST intervention alone is.

To properly control and coordinate harmony of landing movements, aerials need to have sufficient core stability, which allows them to maintain technical elements of large amplitude (Liang, 2014; Esteban-Garcia et al., 2021). Therefore, this trial was designed to determine whether an 8 weeks training intervention of trunk dominant CST would improve landing kinetics in aerials athletes.

## Materials and methods

### Study design

A randomized, controlled, single-blind design was used in this study. A quasi-experimental intra- and inter-subject design with pre-and post-test, and with a control group, was used to identify the effects of 8 weeks of core stability training on the performance of aerials landing. Subjects were randomized into two groups: a control group (CG) or a training group (TG).

### Participants

A total of 18 aerials athletes were randomly divided into two groups: CG (*n* = 4males + 5females; age 15.89 ± 1.54 years; height 163.11 ± 6.19 cm; weight 55.33 ± 5.07 Kg) and TG (*n* = 4males + 5females; age 16.11 ± 2.47 years; height 161.56 ± 5.25 cm; weight 57.56 ± 8.11 Kg). Before the test, the subjects were screened for injuries to confirm that there was no history of core muscle injury, and also strenuous exercise was prohibited within 24 h before the test. The aerials of both groups continued their aerials training regularly, and core stability training was only applied to the TG group. The researcher specified the training program (aerials and core stability), which was executed by a professional trainer (Table 1). To ensure the implementation of the program, the researcher monitored the entire training process. The inclusion criteria for the participants were 6 years of training experience, participation in national or international competitions, and 25 h of training per week. Prior to the start of the study, all athletes and their parents were aware of specific information about the study and provided written informed consent.

**TABLE 1:** Core stability training exercise.

Serial number	Movement
1	Contralateral Support Abdominal Bridge
2	Single Leg Support Glute Bridge
3	Side Bridge
4	Swiss Ball Belly Bridge
5	Swiss Ball Back Bridge
6	V-up
7	Back Hyperextension
8	Seated Swiss Ball Diagonal Pull down
9	Seated Swiss Ball Incline Pull Up
10	Half-Kneeling Side-Twist Throw
11	Torso-twist
12	Standing Dead Bug Shoulder Push
13	Seated Swiss Ball Single-Leg Support Single Arm Dumbbell Shoulder Press
14	Air Resistance Special Pressure Arm Simulation
15	Kneeling Swiss Ball Double Arm Dumbbell Lateral Raise
16	Kneeling Swiss Ball Double Arm Dumbbell Front Raise
17	Barbell Clean
18	Barbell Single Leg Deadlift
19	Asymmetric Weight Overhead Lunge
20	Swiss Ball Single-Leg Squat
21	Step on the Balance Plate, Raise the Bell, and Squat
22	Supine Hip Flexion with One Foot on Foam Roller
23	Standing One Foot on Soft Couch with Eyes Closed
24	Standing One Foot on Soft Couch and Side Toss
25	Jump Off the Box with One Foot

### Procedures

Prior to the measurement, the researcher calibrated the instrument and asked the participants to sign an informed consent form. Then athletes performed a 15 min warm-up and then were familiarized with the balance, isometric muscle strength, endurance and test instrument and methods. The balance was measured on a portable HUR Smart balance (Finland). The trunk isometric tests were measured on the DAVID spine isometric strength testing system (Germany). Core endurance tests were measured on a power lift training station or a yoga mat.

**FIGURE 1:**
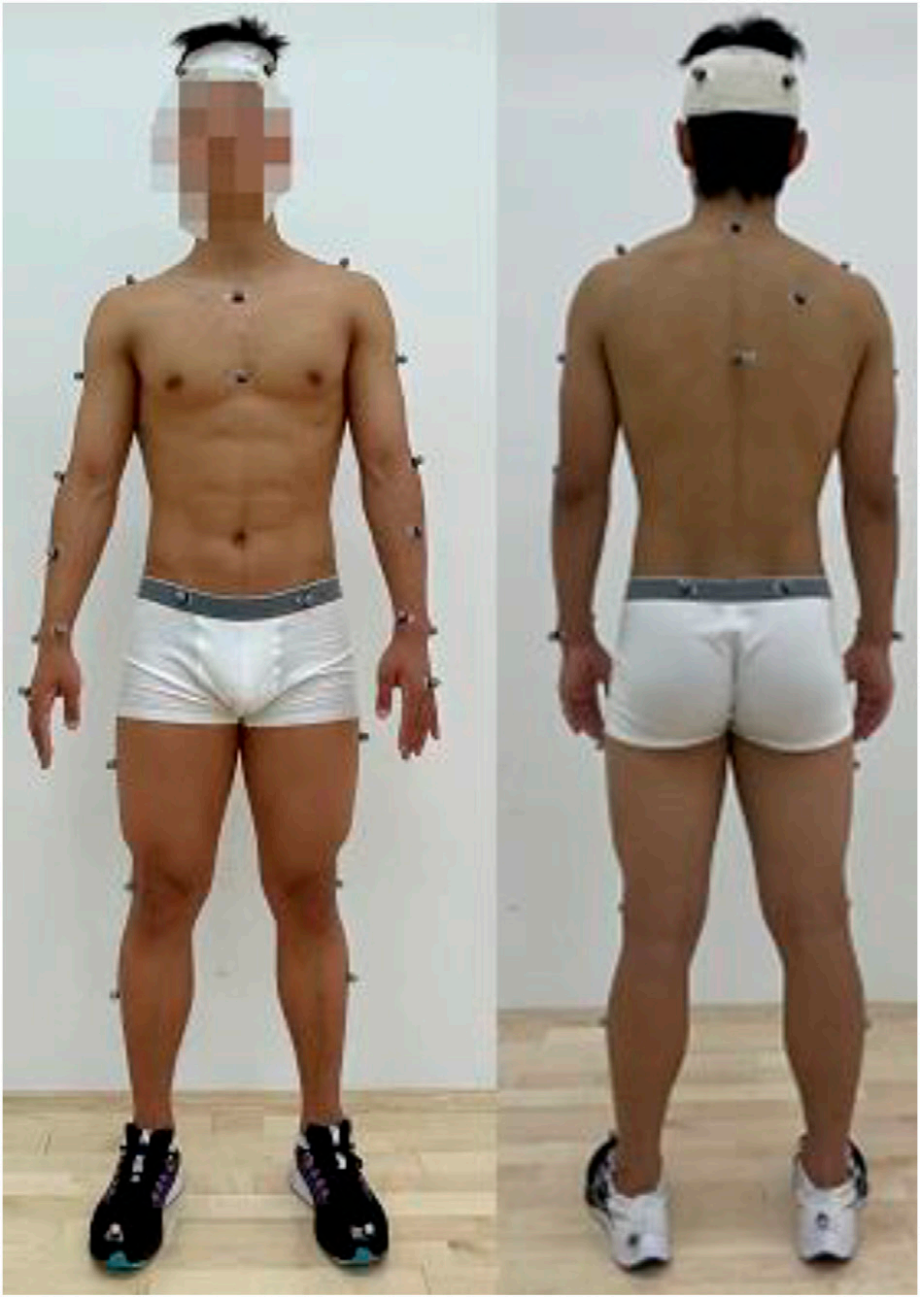
Marker points position.

Landing kinetics text was implemented in the Laboratory of Technical Diagnosis and Skill Assessment of General Administration of Sport of China, Kistler force plate sampling frequency of 1,000 Hz and Vicon Motion System motion capture system sampling frequency of 100 Hz were used. The Marker points were placed in the following positions: head, C7, T10, sternal stalk, glabella, right scapula, acromion, thigh, medial elbow, lateral elbow, forearm, medial wrist, lateral wrist, end of the metacarpal, anterior superior iliac spine, posterior superior iliac spine, thigh, lateral knee, calf, medial ankle, lateral ankle, metatarsal, and heel. To reduce experimental error, the same brand and model of shoes were used in this study, with differences in sizing only (Figure 1).

### Data analysis

SPSS 26.0 software was applied for data processing and analysis. All hypothesis tests were performed using a two-sided test with a significance level of 0.05. An independent sample *t*-test was used for comparison between groups; paired sample *t*-test was used for the analysis of the magnitude of the pre-and post-test elevation, and a nonparametric test was used for non-normally distributed data.

### Intervention

Core stability training was performed twice a week for 8 weeks as a complement to skills training. The core stability training program comprised 25 exercises.

## Results

### Body shape

Results for body shape are shown in Table 2. We observed no difference between the two timelines measured between the two groups (*p* > 0.05). Within-group analysis of TG between pre and post-core stability training showed an increase in waist circumference (t = −2.333, *p* = 0.020).

**TABLE 2:** Body shape results (Mean ± SD).

	Training Group (*n* = 9)		Control Group (*n* = 9)	
	Pre	Post	Pre	Post
Height (cm)	161.22 ± 5.02	161.56 ± 5.25	162.89 ± 6.33	163.11 ± 6.19
Trunk Length (cm)	59.78 ± 7.08	60.00 ± 7.21	61.89 ± 8.18	62.00 ± 8.03
Chest Circumference (cm)	87.22 ± 4.92	87.56 ± 5.13	85.56 ± 4.28	86.00 ± 4.47
Waist Circumference (cm)	68.56 ± 2.24	69.33 ± 2.65^a^	69.22 ± 2.91	69.56 ± 2.83
Weight (kg)	57.78 ± 8.04	57.22 ± 7.33	56.33 ± 5.41	56.00 ± 5.43
Quetelet Index	357.46 ± 39.63	353.34 ± 34.51	345.50 ± 26.40	342.89 ± 25.28

^a^ Indicates the comparison between pre and post-experimental, where * indicates a significant difference, *p* < 0.05; ** indicates a highly significant difference, *p* < 0.01; # indicates the comparison between training group and control groups, where # indicates a significant difference, *p* < 0.05; ## indicates a highly significant difference, *p* < 0.01.

### Performance in core stability

Results for Performance in core stability are shown in Table 3. We observed differences between the two groups for post measurements. Group analysis shows that TG is higher than CG in squat (t = 2.187, *p* = 0.044), trunk lateral bending isometric test (t = 2.187, *p* = 0.044), trunk rotation isometric test (t = 2.167, *p* = 0.046), side bridge (t = 2.140, *p* = 0.048), L control (t = 3.287, *p* = 0.005), single foot triple jump (t = 3.215, *p* = 0.005), single leg balance with eyes closed (t = −2.521, *p* = 0.031). Within-group analysis of TG showed an increase in squat (t = −4.082, *p* = 0.004), trunk flexion isometric test (t = −4.150, *p* = 0.003), trunk lateral bending isometric test (t = −2.668, *p* = 0.008), trunk rotation isometric test (t = −2.666, *p* = 0.008), side bridge (t = −2.666, *p* = 0.008), back hyperextension (t = −4.116, *p* = 0.003), single foot triple jump (t = −4.184, *p* = 0.003), and single leg balance with eyes closed (t = 4.167, *p* = 0.003). Within-group analysis of CG showed an increase in squat (t = −5.068, *p* = 0.001), side bridge (t = −2.310, *p* = 0.021), back hyperextension (t = −2.850, *p* = 0.021), and decrease in L Control (t = −2.616, *p* = 0.031).

**TABLE 3:** Performance in core stability results (Mean ± SD).

	Training Group (*n* = 9)		Control Group (*n* = 9)	
	Pre	Post	Pre	Post
Squat (kg)	99.59 ± 22.48	111.35 ± 28.16**#	82.20 ± 15.03	86.89 ± 18.23**
Trunk flexion isometric test (Nm)	140.11 ± 32.69	152.11 ± 31.07**	118.56 ± 29.48	125.89 ± 39.85
Trunk extension isometric test (Nm)	233.00 ± 80.49	264.67 ± 66.38	218.33 ± 55.77	252.33 ± 107.60
Trunk lateral bending isometric test (Nm)	144.22 ± 33.46	204.28 ± 49.61**#	149.56 ± 34.44	156.33 ± 43.20
Trunk rotation isometric test (Nm)	101.00 ± 44.58	155.56 ± 40.77**#	90.11 ± 30.58	110.06 ± 48.02
10V-up(s)	7.41 ± 0.94	7.08 ± 0.66	7.79 ± 0.82	7.40 ± 0.33
Side bridge(s)	186.30 ± 59.97	236.48 ± 40.35**#	170.67 ± 54.40	192.64 ± 46.34*
L Control(s)	31.53 ± 7.22	41.76 ± 9.52##	32.81 ± 6.17	26.05 ± 10.72*
Back hyperextension (s)	73.02 ± 12.18	83.99 ± 13.31**	66.77 ± 17.30	75.68 ± 17.68*
Single foot triple jump(m)	6.12 ± 0.59	6.64 ± 0.49**##	5.91 ± 0.80	5.91 ± 0.47
Single leg balance (mm)	6.08 ± 1.10	5.88 ± 0.98	6.31 ± 1.37	6.36 ± 0.60
Single leg balance with eyes closed (mm)	9.46 ± 1.15	7.78 ± 0.44**#	9.32 ± 1.33	8.93 ± 1.29

### Performance in landing kinetics

Results for landing time are presented in Table 4. We observed no differences between the two groups for either of the two time-line measurements (*p* > 0.05).

**TABLE 4:** Landing time parameters (ms).

	Training Group (*n* = 9)		Control Group (*n* = 9)	
	Pre	Post	Pre	Post
First peak time	15.44 ± 13.24	16.11 ± 9.36	14.89 ± 5.64	17.56 ± 11.31
First trough time	23.22 ± 24.05	23.78 ± 16.45	21.33 ± 7.6	28 ± 18.04
Second peak time	53.44 ± 26.82	46.22 ± 19.68	52.11 ± 18.58	50.89 ± 23.23
Second trough time	89.33 ± 34.87	81 ± 21.66	93.67 ± 31.89	90.56 ± 29.72
Total time	413.33 ± 140.36	343.33 ± 79.06	508.89 ± 301.47	370 ± 58.52

Results for vertical force gradient are shown in Table 5. We observed differences between the two groups in either of the two time-line measurements. Group analysis of the TG showed higher CG in the First trough/Phase (t = 2.562, *p* = 0.021) and End/Phase (t = 3.698, *p* = 0.002). Within-group analysis of the TG showed an increase in post measurements in End/Phase (t = −4.015, *p* = 0.004), and CG showed a decrease in First trough/Phase (t = 3.201, *p* = 0.013).

**TABLE 5:** Vertical force gradient parameter (N/ms).

	Training Group (*n* = 9)		Control Group (*n* = 9)	
	Pre	Post	Pre	Post
First peak/Phase	114.86 ± 74.96	76.46 ± 27.21	95.63 ± 27.4	74.24 ± 30.26
First trough/Phase	43.34 ± 15.7	43.27 ± 14.89#	44.55 ± 10.08	28.67 ± 8.41*
Second peak/Phase	55.41 ± 21.09	54.37 ± 17.15	69.08 ± 24.61	51.55 ± 18.10
Second trough/Phase	7.59 ± 4.52	8.00 ± 2.52	6.85 ± 2.59	7.24 ± 2.13
End/Phase	0.63 ± 0.24	0.94 ± 0.18**##	0.52 ± 0.19	0.61 ± 0.20

Results for the Pressure center are shown in Table 6. We observed differences between the two groups for post-measurements. Group analysis of the TG showed higher than CG in sagittal axes (t = 2.348,*p* = 0.032), frontal axis (t = 2.441,*p* = 0.034). CG is lower than TG in sagittal axes (t = 2.201, *p* = 0.043). Within-group analysis of the TG showed a decrease in post measurements in the sagittal axes (t = −4.598, *p* = 0.002), and frontal axis (t = 3.116, *p* = 0.014).

**TABLE 6:** Pressure center parameters (mm).

	Training Group (*n* = 9)		Control Group (*n* = 9)	
	Pre	Post	Pre	Post
Sagittal axes	332.63 ± 65.45	244.95 ± 64.47**#	250.47 ± 90.88#	318.75 ± 68.82
Frontal axis	508.88 ± 61.71	392.42 ± 95.6*#	512.82 ± 88.96	475.83 ± 37.05
Horizontal axis	715.84 ± 42.47	709.03 ± 45.16	716.49 ± 35.4	700.24 ± 30.75

Results for Hip, knee, and ankle range of motion are shown in Table 7. We observed differences between the two groups for post-measurements. Group analysis of the TG showed higher than CG in peak power hip changing range (t = 2.666, *p* = 0.017), Peak power knee changing range (t = 2.256, *p* = 0.049). Within-Group analysis of the CG showed a decrease in peak power knee range (t = −2.560, *p* = 0.034).

**TABLE 7:** Hip, knee, and ankle range parameters (°).

	Training Group (*n* = 9)		Control Group (*n* = 9)	
	Pre	Post	Pre	Post
Peak power hip range	141.28 ± 10.92	142.99 ± 10.31	143.99 ± 10.95	150.37 ± 6.41
Peak power hip changing range	7.62 ± 2.26	8.21 ± 1.94#	7.41 ± 2.02	5.79 ± 1.91
Peak power knee range	147.89 ± 10.21	152.2 ± 5.92	147.11 ± 9.77	157.28 ± 12.33*
Peak power knee changing range	15.41 ± 4.27	17.46 ± 1.88#	15.98 ± 5.98	12.89 ± 5.77
Peak power ankle range	82.67 ± 4.94	86.65 ± 4.03	92.25 ± 26.09	83.92 ± 5.79
Peak power ankle changing range	14.56 ± 5.08	18.8 ± 4.08	17.31 ± 6.15	21.21 ± 4.71

## Discussion

The purpose of this study was to analyze the effects of 8 weeks of core stability training on athletes who were still training in aerials on body shape, performance in core stability, and performance in landing kinetics. The main findings were that the core stability training evoked improvements in waist circumference, squat, trunk lateral bending isometric, trunk rotation isometric, side bridge, L control, single foot triple jump, single leg balance with eyes closed, sagittal axes pressure center, frontal axis pressure center, peak power hip changing range, and peak power knee changing range.

### Body shape

In terms of body shape, TG showed higher waist circumference after core stability training. As far as we know, there have been no studies on the effect of core stability training on the body shape of aerials. It is well known that resistance training is considered the optimal method to increase muscle circumference (Fisher et al., 2013), aerobic training is the main training method to reduce body fat (Muscella, 2020), improve the antioxidant capacity of the body (Brooks et al., 2008), and improve cardiorespiratory fitness (İşleyen and Dağlioğlu, 2020), and high-intensity interval training is often studied in comparison with moderate-intensity continuous training (O'Brien et al., 2020), which is one of the most popular training methods in recent years and is an effective means of rapid fat loss by increasing the metabolic rate (Maillard et al., 2018). There are different views on the effects of CST on body shape. Mehdizadeh, (2015) found that CST improved weight, BMI, and waist circumference in postmenopausal menopausal women (*p* = 0.03), as well as a highly significant effect on total plasma cholesterol levels (*p* = 0.007), but not on lipoproteins and other components. In contrast, Segal et al. (2004) performed Pilates training on 32 subjects for 8 weeks and found no significant changes in height, weight, and body fat, but significant improvements in flexibility. Therefore, although CST may already have a positive effect on the body shape of aerials, only TG waist circumference increased significantly, other indicators was no significant change.

### Core stability performance

Previous studies on the effects of CST on performance have conflicting results. Sharma et al. (2012) improved snap and jump height in volleyball players through 9 weeks of CST. Nesser et al. (2008) found that CST improved core strength and sprint speed in soccer players. In contrast to these studies, Stanton et al. (2004) used a Swiss ball to perform CST on 18 track and field athletes for 6 weeks and found a significant increase in CS (*p* < 0.05), but no significant differences in electromyographic activity of the core muscle groups, running economy, or running posture. Reed et al. (2012) also pointed out in a systematic review that CST is generally effective in improving the specific athletic ability of professional athletes, and that CST is only a part of physical training for professional athletes, so CST arrangement must emphasize the combination of CST and specific athletic characteristics. The main reason for the conflicting results is that researchers have overemphasized CST, thereby neglecting other training methods as well as specificity (Hibbs et al., 2008; Okada et al., 2011).

This study found an extremely significant increase in a squat in both groups after 8 weeks of CST and a significantly higher TG than CG. Consistent with the results of previous studies, Dello (Dello Iacono et al., 2016) found that CST significantly increased soccer players’ peak power of knee flexion and extension and improved the symmetry of the legs. Trunk strength is commonly used in sports and rehabilitation as an important indicator of CS, and isometric muscle strength tests are considered the gold standard for trunk strength (Behm et al., 2005; Cowley et al., 2009; Kocahan and Akinoglu, 2018). In this study, the subjects were tested for trunk isometric strength using the DAVID spinal rehabilitation system. The results revealed that the trunk flexion, lateral bending, and rotation isometric muscle strength were significantly higher in the TG than in the pre-measurements. Previous studies have found that the stronger the core strength of the aerials, the better the static balance of the body and the higher the stability of the landing (Yantao, 2010).

The 10 V-up finish time was slightly reduced, indicating that CST has less influence on the rapid contraction ability of core muscle in aerials. The side bridge, L Control, and back hyperextension are indicators of endurance of the core muscle. In this study, we found that side bridge, L control, and back hyperextension were all improved to different degrees in TG, and side bridge and L control were significantly higher than CG, CST can effectively improve the endurance of the core muscle. The results are in agreement with previous studies (Sandrey and Mitzel, 2013) and have a good effect on the treatment of chronic lower back pain (Shamsi, 2016). Waldhelm and Li, (2012) concluded that core endurance is the most reliable indicator of CS, in order of endurance > flexibility > strength > neuromuscular control > functional movement screening.

This study found that CST also improved explosive power and proprioception. Single foot triple jump can reflect the explosive power and land cushioning ability and is also often used to evaluate the symmetry of legs for athletes (Ming and Xuesong, 2022), which is an important index for preventing sports injuries and evaluating rehabilitation effects. In this study, we found that the TG single-foot triple jump was higher than pre-measurement and CG, and there was no difference between the CG for either of the two time-line measurements. The single-leg balance with eyes closed showed that TG was better than CG. Hutt and Redding, (2014) pointed out that the closed-eye dance exercises led to the improvement of ballet dancers’ star balance test scores, which was beneficial for ballet dancers to fight against the interference of stage lighting and perfect their performances, and encouraged ballet dancers to try closed-eye training in their daily dance classes. McNitt-Gray et al. (2001) found in their study of gymnasts’ aerials flip movements that the angular velocity of the thighs was faster than the hips during the front flip and slower than the hips during the backflip, reflecting the limiting difference in the direction of the flip on vision, higher difficulty movements are more visually intrusive than simple difficulties. The improvement of balance ability in the closed-eye environment is conducive to the aerials to adapt to the changing outdoor environment and land stably after completing difficult aerials flip movements, making the athletes’ movements more stable.

### Landing kinetics

According to the momentum theorem (N-G)▪△t = △MV(Yantao, 2012), when the human body falls from different heights will produce the corresponding momentum, when the momentum growth and △MV are certain, the weight of the body is constant, △t and N is inversely proportional, the longer action time the smaller the reaction force with better cushioning effect. In this study, the landing time in both groups was shortened after CST, but there was no significant difference. It indicates that both CST and strength training can improve trunk and lower limb rigidity to complete cushioning in a shorter action time.

The vertical force gradient parameter is an index that reflects the average rate of change of force values per unit of time, dF/dT is defined as the gradient of force (Yantao and Yi, 2021), and a big gradient of force values indicates a good explosive force (Yongdian et al., 2002). Vertical force gradient changed after CST. In the first wave peak/phase, the two groups had almost the same value, which was caused by the cushioning action, and rapid transition from forefoot to all foot, so the force value gradient was the same with no difference in average weight, with the forefoot landing first for cushioning. The first trough/phase of TG was significantly higher than CG, indicating that TG cushioning was more adequate and more stable in contact with the ground, which improved the rate of change of impact load (Farley et al., 1998). Ending moment/time phase, TG was significantly higher than CG and pre-measurement, and this result was influenced by the landing total time, it could be found that TG was smaller than CG, indicating that TG was able to complete a high-quality landing buffer in a shorter time. In addition, the gradient fluctuation of TG force change is smaller than CG, which indicates that the up and down fluctuation of force during the landing buffer is small, the impact force by the human body is more moderate, and the buffering effect is better than CG.

The pressure center reflects the body’s ability to maintain balance and resist tipping. Pressure center displacement, especially in the front-back and left-right directions, indicates poor control of landing stability because when the gravity is displaced, it inevitably causes complications in human control. This study found that the pressure center in the front-back and left-right directions of the TG was significantly lower than pre-measurement and CG. The increased displacement of gravity in the front-back directions of the CG. When the control of the gravity in the left-right directions is poor, it is compensated by increasing the displacement of gravity in the front-back directions to make the landing control easy, which is consistent with the results of previous studies (Yantao and Yi, 2021).

When the human body falls to the ground, it usually adopts the posture of hip flexion, knee flexion, and ankle extension for cushioning, which aims to reduce the impact force of the ground by prolonging the landing time, also When the joint is in flexion and extension it will reduce the impact force and avoid joint injury (Neumann, 2010). There was no significant difference in the peak power hip, knee, and ankle range during landing between the two groups, but there is a tendency for the flexion angle to decrease. Tsai et al. (2020) performed CST on 16 volleyball players and found that after CST the athletes increased hip flexor strength by 19%, hip external rotation strength by 14%, knee flexor strength by 25%, knee extension strength by 24%, decreased trunk flexion angle at landing by 6.5°, and decreased knee flexion angle by 9.5°, concluded that increased core strength resulted in decreased trunk flexion angle in landing. The hip and knee changing range of TG were significantly greater than CG. TG actively reduces the impact force of the ground on the human body by increasing the joint changing range, and this landing method was more stable. Devita and Skelly, (1992) found that the angle of knee flexion during landing classified landings into soft landings (<117°) and rigid landings (>117°), with rigid landings producing greater hip extension force (*p* < 0.01) and knee flexion force (*p* < 0.01) and more upright trunk postures than soft landings, while soft landings were able to absorb 19% more ground reaction forces than rigid landings, with less impact on the muscles and bones.

## Conclusion

Our results suggest that combining a traditional aerials program with a core stability training program could lead to increased strength, endurance, balance, and proprioception. In addition, core stability training can improve landing stability.

## Data Availability

The original contributions presented in the study are included in the article/Supplementary Materials, further inquiries can be directed to the corresponding authors.
